# The Emerging Role of RNA in Diseases and Cancers

**DOI:** 10.3390/ijms24076682

**Published:** 2023-04-03

**Authors:** Alessandro Fatica

**Affiliations:** Department of Biology and Biotechnology ‘Charles Darwin’, Sapienza University of Rome, 00165 Rome, Italy; alessandro.fatica@uniroma1.it

In recent years, there has been a growing interest in the role of RNA in diseases and cancers. It is now becoming increasingly clear that non-coding RNAs, such as microRNAs, long non-coding RNAs (lncRNAs), and circular RNAs (circRNAs), play an active and crucial role in regulating gene expression and cellular functions. One area where the role of regulatory RNA is particularly prominent is in cancer research. Furthermore, the RNA modification field, also known as epitranscriptomics, has recently gained attention due to research into the regulation of gene expression and cellular functions. These modifications involve the addition, removal, or alteration of chemical groups on RNA molecules, and are known to impact RNA maturation, stability, and translation. Recent evidence suggests that RNA modifications also play a critical role in various diseases and cancers.

This Special Issue of the *International Journal of Molecular Sciences*, entitled “The Emerging Role of RNA in Diseases and Cancers”, includes a total of nine contributions: one research article, one case report, and seven reviews providing new information about the role of non-coding RNAs and RNA modifications in normal and pathological conditions. 

Researchers have discovered that certain types of regulatory non-coding RNA, such as microRNAs and lncRNAs, can control cell growth and differentiation by regulating key signaling pathways. These RNAs can act as either oncogenes or tumor suppressors, depending on the specific context. Ciafrè et al. thoroughly reviewed the important role of lncRNAs in cancer stem cells, the different mechanisms through which they work, and how their modulation can affect tumorigenesis [1]. On the same topic, Giuliani et al. reviewed the different mechanisms of lncRNA function that have been identified in the context of breast cancer [2].

In addition to cancer, RNA has also been implicated in a variety of other diseases, including neurological disorders, cardiovascular diseases, and viral infections. For example, mutations in RNA-binding proteins have been linked to amyotrophic lateral sclerosis (ALS) and frontotemporal dementia (FTD). Colantoni et al. investigated the effect of mutation in the RNA-binding protein FUS, which characterizes familiar forms of ALS, on the formation of circRNAs in in vitro-derived human motoneurons [3]. The study also analyzed the potential function of deregulated circRNAs to act as microRNA competitors, with possible implications for ALS pathogenesis. Moreover, the review by Silvestri et al. provided a summary of the role of RNA-binding proteins in the nervous system and their involvement in ALS, Parkinson’s disease (PD), and Alzheimer’s disease (AD) [4]. 

The emerging role of RNA in diseases and cancers has significant implications for diagnosis, prognosis, and treatment. RNA-based biomarkers can provide important information about disease progression and treatment response, while RNA-targeted therapies, such as RNA interference (RNAi) and antisense oligonucleotides (ASOs), hold great promise for treating a wide range of diseases. However, there are also significant challenges to be overcome in translating RNA-based research into clinical practice. For example, RNA-based therapies face issues related to delivery and specificity, as well as potential off-target effects. Additionally, the development of RNA-based biomarkers requires careful validation and standardization across different platforms and patient populations. Garbo et al. reviewed the benefits and the limits of RNA therapeutics by describing the non-coding RNA molecules that are being tested in clinical trials, and by analyzing the delivery strategies for different therapeutical applications [5]. Rincon-Riveros et al. reported an interesting case study in which the authors utilized comparative transcriptome analysis for the diagnosis of primary breast angiosarcoma, a rare cancer representing less than 0.05% of breast cancer cases [6]. This study highlighted the use of RNA expression to predict an immune and inflammatory response in patients.

Lee et al. examined the role of microRNAs as biomarkers and therapeutic targets for muscle dystrophy, such as Duchenne muscular dystrophy (DMD) and Becker muscular dystrophy (BMD) [7].

One of the most well-studied RNA modifications is N^6^-methyladenosine (m^6^A), which is the most prevalent modification found in eukaryotic mRNA. m^6^A has been implicated in cancer development and progression, with dysregulated m^6^A modification patterns associated with poor prognosis in several types of cancer. Studies have shown that m6A modification can affect mRNA stability, translation, and alternative splicing, which, in turn, can impact cell growth, proliferation, and survival. Fernandez Rodriguez et al. analyzed the role of m^6^A in the regulation of the global rate of mRNA translation and the selective translation of specific mRNAs in cancer [8]. Furthermore, Cesaro et al. reviewed the function of the recently identified N^6^,2′-O-dimethyladenosine (m^6^A_m_) modification by describing the methodologies for its mapping, the enzymes responsible for m^6^A_m_ regulation, and their impact in gene expression regulation. Furthermore, they discussed the emerging roles of m6Am in tumorigenesis [9].

The emerging role of RNA in diseases and cancers represents a major shift in our understanding of the molecular mechanisms underlying complex diseases, such as cancer. By continuing to explore the complex roles of RNA in cellular processes, we can pave the way for the development of new diagnostic tools and therapeutic approaches that could ultimately improve outcomes for patients with a wide range of diseases.
